# Non-foraging tool use in European Honey-buzzards: An experimental test

**DOI:** 10.1371/journal.pone.0206843

**Published:** 2018-11-21

**Authors:** Carlos Camacho, Jaime Potti

**Affiliations:** Department of Evolutionary Ecology, Estación Biológica de Doñana–CSIC, Seville, Spain; Indian Institute of Science, INDIA

## Abstract

Examples of tool-use behaviors by birds outside foraging contexts are scarce and limited to a handful of species. We report a field experiment aimed to test whether an observed suite of odd behaviors by European Honey-buzzards (*Pernis apivorus*) represents use of green twigs cut from trees and woody shrubs as a tool to attract ants for anting. Specifically, we tested whether buzzards are selective in their choice of twigs, under the assumption that birds would prefer easy-to-collect twigs from plants that effectively attract ants. Experimental results lend support to our hypothesis that European Honey-buzzards cut green twigs of Montpellier maple trees (*Acer monspessulanum*) and, to a lesser extent, of Pyrenean oaks (*Quercus pyrenaica*) for their immediate use as ant attractors. Fresh twigs of both tree species attracted large numbers of ants, suggesting that their preferential use in the reported behavior of Honey-buzzards is not a random selection of the available plant material. Maple twigs, however, were the easiest to break and oak twigs the hardest compared to other plants in the community. This suggests that the relative ease of cracking of maple twigs may account for the preference Honey-buzzards have for this plant species as compared with Pyrenean oak, whose twigs demand considerable more effort from the birds to break. Our results lend support to the inclusion of the reported behavioral sequence by this raptor species as a potential example of tool use in birds outside the usual foraging context.

## Introduction

The neurological, ecological, and evolutionary correlates of tool use by birds are a thriving area of research [1–5]. Several bird species are known to use tools to facilitate access to food resources. For instance, New Caledonian crows (*Corvus moneduloides*) are famous for using different types of hook tools to capture prey [6], woodpecker finches (*Cactospiza pallida*) use twigs or cactus spines to pry arthropods out of tree holes [7], and burrowing owls (*Athene cunicularia*) use dung as a bait to attract the beetles they feed on [8]. Examples of bird tool use outside a foraging context (e.g. use of bark-wad as a paintbrush by satin bower-birds (*Ptilonorhynchus violaecus*) [9] and use of leaves as insect repellent by Darwin’s finches [10]) are however very scarce and limited to a handful of species.

In 2012, we observed a European Honey-buzzard (*Pernis apivorus*) in a forest area of central Spain performing a complex suite of behaviors never reported before. The buzzard collected and spread fresh twigs–mostly maple and, to a lesser extent, oak twigs–along a sandy road, and then stretched its wings full-length on the ground (see S2 Fig and [11] for pictures and a detailed description of the behavioral sequence). This behavior was repeated by one single individual for at least 13 days in a row on a sun-drenched slope. Based on postural similarities to other bird species known to grab ants and rub them into their feathers (i.e. anting behavior; [12, 13]), we hypothesized that through this behavior, the bird may have been attracting ants to remove parasites, although such a repetitive display for relatively long periods has not been previously reported in an anting context [11].

Based on recent definitions, anting is accepted as a form of tool use [6, 14]. Following Alcock [15], tool use is here defined as a behavior involving “the manipulation of an inanimate object, not internally manufactured, with the effect of improving the animal’s efficiency in altering the form or *position* of some separate object” (our italics). The behavioral sequence performed by the Honey-buzzard fulfils Alcock’s tool use definition by both the use of an inanimate object, not internally manufactured by the bird–the set of twigs–and, hypothetically, altering the position of a third party–the ants–through the attraction by the ‘lure(s)’. The latter may consist either of volatile substances released from the twig wounds [16, 17] or of extrafloral nectaries highly attractive to ants [18]. Due to the shyness of the buzzard–which forced us to observe from afar with binoculars–we could not directly attest if ants climbed into the bird’s plumage. In addition, we have been unable to accumulate additional observations of the described behavior, even when intensive fieldwork in other research projects in the same area has continued for six years in a row (2013–2018), and European Honey-buzzards have been regularly seen in all years. This is hardly surprising, however, as our original observations were serendipitously facilitated by the substrate where the behavior took place: an open, clear-grounded space where the regularity of the twig setting was evident to the human eye. Therefore, the hypothesis that Honey-buzzards may be deliberately trying to attract ants through the collection of fresh twigs remains to be confirmed.

Here we report the results of a field experiment that aimed to test the suitability of different tree and woody shrubs to attract ants. If we find the buzzard selects twigs from plants that are attractive to ants, it would support the hypothesis that the observed tool use behavior serves to attract ants for anting. This would be an additional example of tool use in birds outside the usual foraging context.

## Methods

### Ethics statement

The Consejería de Medio Ambiente (Comunidad de Madrid) granted the research permits for fieldwork in La Hiruela (Madrid) between 1984 and 2018. No specific permissions were required for this research, and we confirm that the field experiments did not involve endangered or protected species.

### Study area

Fieldwork was conducted between 30 June and 10 July 2013, the year after the behavioral sequence was observed [11], in a mature oak forest of 9.3 ha located near La Hiruela, about 80 km northeast of Madrid (41°04'N 3°27'W; 1,250 masl). No other records exist for the reported behavior in this area either before or after we observed it in 2012. European Honey-buzzards are long-distance migrants that occur in the area at very low densities (2 pairs/10^5^ ha) between May and September (Regional Government of Castilla-La Mancha, unpubl. data). The vegetation of the study site is dominated by Pyrenean oaks (*Quercus pyrenaica*) at a mean density of 460 trees ha^−1^, with a dense ground cover (0.5−3 m high; mean cover 80%) and understory of oak saplings, laurel-leaved rock rose (*Cistus laurifolius*), hawthorn (*Crataegus monogyna*) and briar root (*Erica arborea*). Montpellier maple trees (*Acer monspessulanum*) are comparatively scarce and restricted to a small area of 3.1 ha near the northeastern edge of the forest. No previous knowledge about the diversity or composition of the ant community is available for the study area.

### Examining the suitability of plants as a tool for anting

First, we estimated the strength needed to break fresh, green twigs from the species most commonly used in the behavioral sequence (maple tree) and three additional (non-herbaceous) species (rock rose, hawthorn, and oak), which were selected according to their higher local abundance compared to other plants. Besides maple (n > 40 twigs collected in 13 days), the buzzard collected oak twigs, but in a much smaller proportion than might be expected from its qualitative abundance (n = 3 twigs collected in 13 days). No cases of rock rose or hawthorn use were recorded throughout the observation period. Green twigs similar in size to those used by the European Honey-buzzard (4–5 mm diameter and 20–25 cm length; [11]) were collected from randomly selected plants using pruning shears. Because this work was conducted while sampling birds breeding in nest-boxes in the same area, to ensure random selection we collected the plants within a 5–10 m radius from the location of the last nest-box sampled each day. No more than one twig sample collected between 0.5 and 2 m above the ground was taken from each plant. Each twig was subjected to a bending test to determine the breaking strength (i.e. ease of cracking). For this, each piece was bent in the middle by pressing one end until it broke, and the fracture angle was measured using a manual goniometer, assuming a negative relationship between the fracture angle and ease of cracking. Even though we could not observe the buzzard collecting green material, during the observation period we found broken twigs on the maple tree adjacent to the place where the bird was seen, suggesting that the buzzard broke green twigs by bending them down. Moreover, although there is no specific information for the study species, direct observations of other raptors using green twigs as nest material indicate that they use their beak to reach up and snip off the twigs while hanging upside-down [19]. Based on these observations, we are confident that the bending test mimics the manner in which buzzards collect green twigs. Overall, 120 reasonably identical samples −30 per species− were subject to the bending test.

Second, we conducted a selection experiment to compare the effectiveness of ant attraction by the focal plant species, measured as the total number of (diurnal) ants attracted. For this, we collected fresh green twigs similar to those collected by the bird (one per plant species, 20–25 cm length) and immediately set them parallel to each other on the ground surface, at a distance of 1 m between them. In addition, we used an inanimate object (i.e. a crumpled 80-g paper of 25 cm in length) as a control to account for the possibility that ants may be attracted by the shade or refuge provided by leaved twigs. One sample of each of the ant species observed walking on the twigs setting (see ‘Results’) was collected and stored in ethanol for subsequent identification. To determine the minimum time required for ants to approach the twigs, we conducted the same experiment as described above and continuously monitored the twigs within 90 min from placement (n = 3 replicates). For all the three replicates, we found that it took between 20–30 min for the first ant to appear, whereas no ants were seen approaching the twigs after 60 min from placement. Based on the results of the pilot experiment, the number of ants was counted after 30, 45, and 60 min since the onset of the trial, and the maximum number of ants found *on* each item at any of the time points was recorded as a measure of ant attraction. Plant selection experiments were conducted between 9:00 and 13:00, when the behavioral activity of the European Honey-buzzard had been highest (pers. obs.). Replicates of the selection experiment were conducted at randomized kilometric points located at a minimum distance of 50 m along the 2.6 km trail crossing the study area. Overall, a maximum of 23 random points could be defined within the 2.6-km trial without violating the minimum distance criterion.

### Data analysis

All statistical analyses were performed in R v.3.3.1 (http://www.R-project.org). Differences in the ease of cracking between plant species were examined with a linear model after log transformation of fracture angle. Plant species (class variable, 4 levels) was fitted as a fixed effect in the model. To examine differences between plant species in the effectiveness of ant attraction, we fitted a Generalized Linear Mixed Model (GLMM, Poisson error distribution) using the R package ‘lmerTest’ [20]. Number of ants was used as the response variable. Plant species (class variable, 5 levels including the control) was included as a fixed effect. In addition, trial identity was included in the model as a random effect to control for the non-independence of twig samples located adjacent (1m) to each other. To assess the overall significance of ‘plant species’ we compared the explanatory power of models including and excluding the fixed effect (i.e. a null model including only the random intercept) with a likelihood ratio test using the function *anova* [21]. Post-hoc Tukey tests were used to evaluate differences between plant species in ease of cracking and attractiveness to ants using the *glht* function in the R package 'multcomp' [22].

## Results

**Fig 1:**
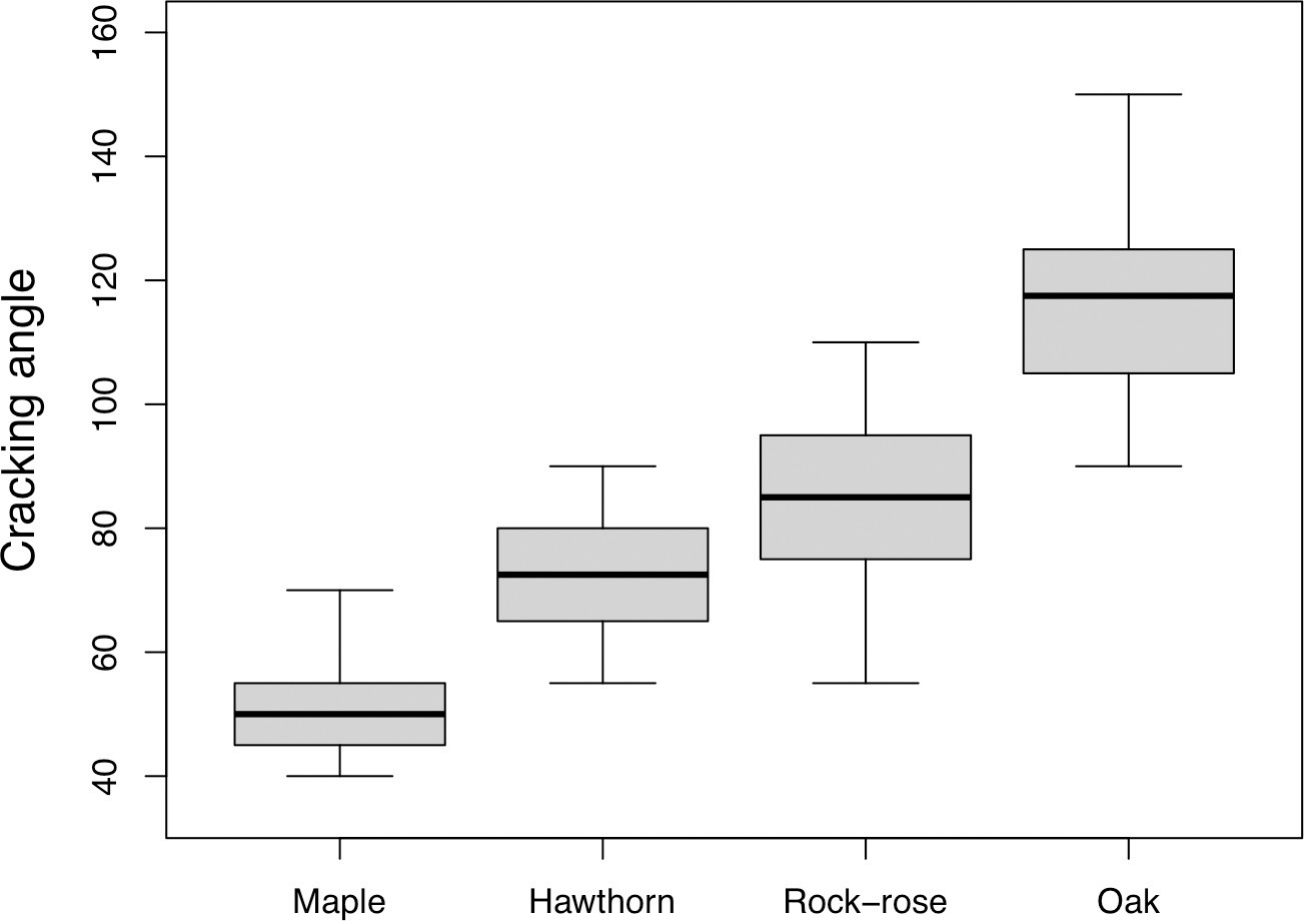
Ease of fracture of fresh twigs. Cracking angles of green twigs of maple tree and of the three most common woody plants in the study area. Shown are the median (central line in box), the upper and lower hinges (edges of the box approximating the first and third quartiles), and the whiskers (defined as 1.5x the hinge spread).

Ease of cracking was highest for maple and lowest for oak, while the other two species showed intermediate fracture angles (Chi = 483.6, d.f. = 3, P < 0.0001; Table 1; Fig 1).

**Table 1:** Results of Tukey tests comparing the cracking angle of green twigs of maple, oak, hawthorn, and rock-rose.

	Estimate	SE	*t*	*P*
Maple—Hawthorn	-0.142	0.016	-8.820	<0.0001
Oak—Hawthorn	0.206	0.016	12.787	<0.0001
Rock-rose—Hawthorn	0.071	0.017	4.265	0.0002
Oak—Maple	0.349	0.016	21.607	<0.0001
Rock-rose—Maple	0.213	0.017	12.850	<0.0001
Rock-rose—Oak	-0.136	0.017	-8.181	<0.0001

**Fig 2:**
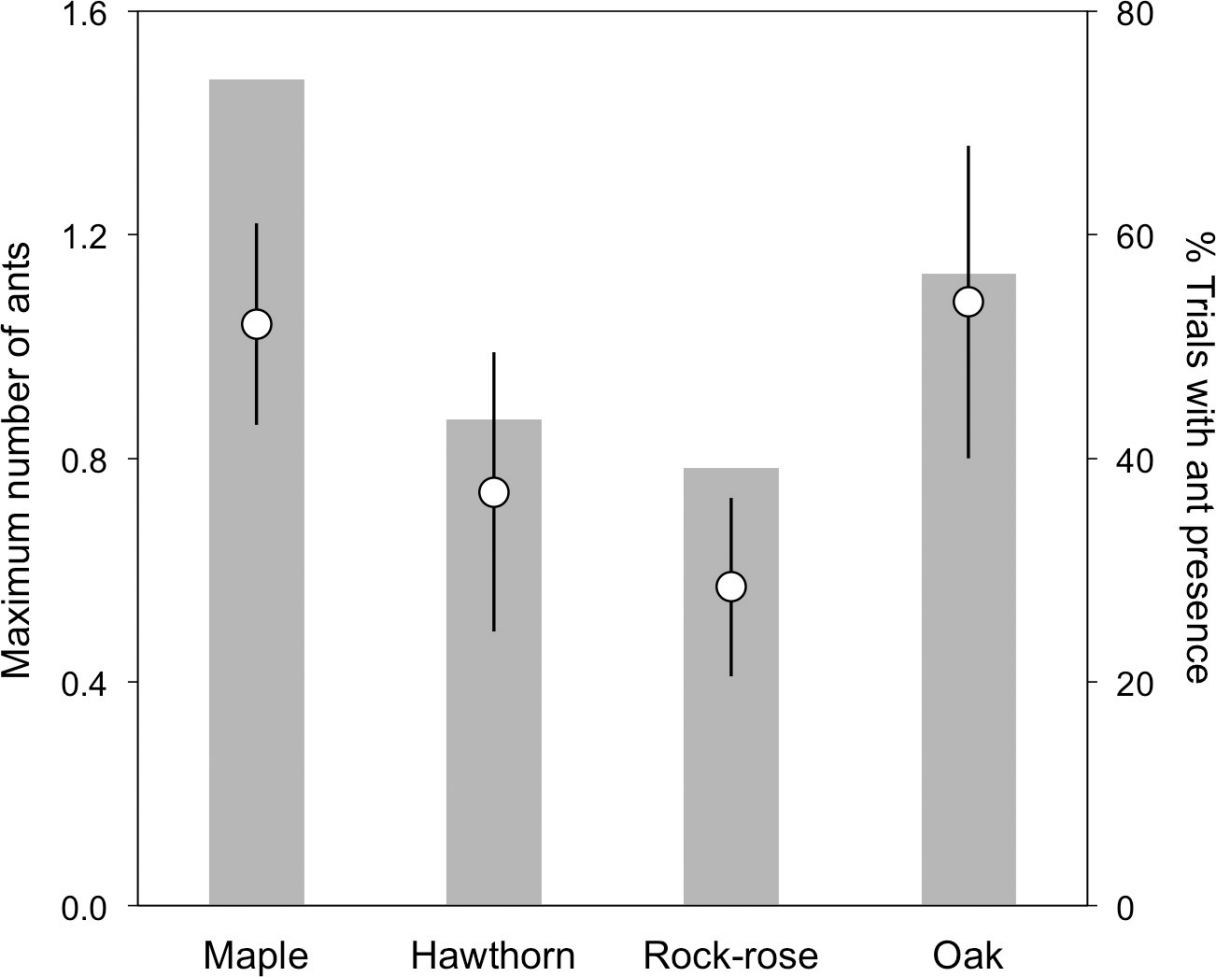
Plant preferences of diurnal ants. Open circles (left y-axis) denote the mean (± SE) of the maximum number of ants observed on each focal species during the trials. Bars (right y-axis) show the percentage of trials in which at least one ant was seen walking on the focal species. Note that the control is omitted from the figure since no ants were ever observed on it.

Results of the selection experiment also revealed significant differences between plant species in the effectiveness of ant attraction (Chi = 16.4, d.f. = 4, P = 0.0026), measured as the number of ants (Table 2, Fig 2). Maple and oak twigs attracted significantly more ants than the control (Table 2, Fig 2). Maple twigs attracted the largest number of ants, followed by oak, hawthorn and lastly rock-rose twigs (Fig 2). However, post-hoc tests revealed no differences among these plants in their attractiveness to ants (Table 2). No ants were ever found on the control paper sheets, whereas fresh twigs attracted at least one ant (*Lasius spp*. and in, only one case, also *Cataglyphis spp*.) in 39–74% of the trials, depending on the plant species (Fig 2).

**Table 2:** Results of Tukey tests comparing the number of ants attracted by the four plant species and the control (paper sheet) used in the selection experiment.

	Estimate	SE	*Z*	*P*
Hawthorn—Control	1.041	0.475	2.193	0.1758
Maple—Control	1.386	0.456	3.037	0.0192
Oak—Control	1.427	0.455	3.139	0.0138
Rock-rose—Control	0.773	0.494	1.567	0.5096
Maple—Hawthorn	0.345	0.317	1.088	0.8073
Oak—Hawthorn	0.386	0.314	1.227	0.7285
Rock-rose—Hawthorn	-0.268	0.368	-0.728	0.9482
Oak—Maple	0.041	0.286	0.143	0.9999
Rock-rose—Maple	-0.613	0.344	-1.780	0.3758
Rock-rose—Oak	-0.654	0.342	-1.912	0.3022

Despite the small size of the twigs used in the experiment, up to 5 ants were sometimes seen on one single twig, and up to 11 ants were simultaneously observed on twigs of different species during successful trials, defined as those where at least one ant was seen within 60 min (20 out of 23 trials). In addition, ant attraction took no longer than 30 min in 80% of successful trials, suggesting that the attractiveness to diurnal ants of recently collected twigs may be high.

## Discussion

Our experimental results lend support to our hypothesis that European Honey-buzzards are selective in their choice of twigs and that they cut and use green twigs of, at least, Montpellier maple trees and Pyrenean oaks as ant attractors. Green twigs of maple and oak, unlike the other two plants tested, attracted more ants than might be expected by chance, suggesting that the preferential use of maple and oak twigs in the reported behavior is not a random selection of the available plant material in the forest. This is much more obvious in the case of maple twigs, which are clearly preferred by the buzzards despite the species being restricted to a small patch [11]. Moreover, we have demonstrated that, among the woody species we tested, maple was the easiest and oak the hardest to break by humans and, presumably, also by the raptor. Taken together, the results of the selection and the bending experiments suggest that ease of breaking and attractiveness to ants interact to influence plant choice by buzzards.

Based on the results of the bending test, it seems that ease of cracking is the key factor determining the preference of buzzards for maple twigs over the other plants. Given the similarity in ant attractiveness between maple and oak twigs, buzzards are more likely to collect maple twigs as a tool for anting because, as shown in this study, oak twigs demand considerably more effort from the birds to break than maple twigs. Hence, according to the interaction between ease of breaking and attractiveness to ants, the attractiveness of different plant species to buzzards appears to be highest for maple tree, followed by oak, hawthorn, and rock-rose.

Experimental and observational evidence strongly suggest that the observed use of twigs serves to attract ants presumably for anting. However, since anting on buzzards has not been observed directly, other possible explanations for this behavior should be considered. For example, European Honey-buzzards could use fresh twigs as a bait to attract insect prey [8]. However, this is unlikely because they primarily feed on larvae of social wasps [23] and no other arthropod was attracted by the twigs. Buzzards might also perform the observed suite of behaviors in mating and social contexts by conveying information on the strength and condition of the performer [24]. However, no other interacting buzzards were ever seen during the observation period and, at any rate, one would expect that, under this hypothesis, the demonstrator would choose twigs larger than only 25 cm and/or the toughest (not the easiest) twigs to break [25].

One needed missing piece of the story is the lack of observations of other individuals performing the presumed anting behavior. However, the literature on anting in birds is dominated by anecdotal observations, as was also in our case, as the behavior is rare despite being taxonomically widespread. Indeed, anting has been described as practiced, either habitually or occasionally, by solitary individuals of about 210 avian species, including at least another diurnal raptor, the Common kestrel (*Falco tinnunculus*) [26–28]. We propose that the issue of tool use by buzzards in relation to anting may be most straightforwardly addressed in captivity, namely in wildlife rehabilitation centers where the staff will be able to easily manipulate the availability of prospective tools and ant abundance while also controlling for the bird’s health. Nevertheless, the possibility exists that the described buzzard behavior may be an innovation of this specific individual and which has not spread to others by imitation or social learning [29].

## Conclusions

The complex behavioral sequence performed by European Honey-buzzards described in this study involved the fabrication and deliberate staging of tools in a setting aimed to attract ants with the purpose of immediately undertaking anting behavior. However, further observations on more individuals of this species confirming that this is for anting would help validate our findings. A question for further research is what function anting behavior could serve in this and other raptor species, which seem to indulge in this behavior very infrequently (e.g. soothing skin irritation during feather molt [30] or controlling feather parasites and combat feather-degrading bacteria and skin infections [31]). Irrespective of what the function is, the suite of Honey-buzzards behaviors leading to anting fit both of Alcock’s criteria [15] for tool use, as the bird *manufactures* particular woody species to obtain twigs or the tools needed to attract ants. Taken together, with the well-known examples of Egyptian vultures (*Neophron percnopterus*) cracking eggs with stones [32], green-backed herons (*Butorides striatus*) using bait to catch fish [33], and few other examples [34] of use of tools to access food, our observations and experiments suggest that the observed behavioral sequence [11] adds to a small but growing body of evidence for tool use by birds.

## Supporting information

S1 Fig European Honey-buzzard (*Pernis apivorus*). Focal bird of the study in central Spain, passing through the oak forest where maple and oak twigs were collected as a tool for anting. Photo: Octavio Jiménez Robles. Printed under a CC BY license, with permission from Octavio Jiménez Robles, original copyright 2012.
(PDF)

S2 Fig Focal bird in a posture consistent with anting. European Honey-buzzard (*Pernis apivorus*) standing on the ground, with partly spread wings and tail. Maple and oak twigs used as a tool to attract ants for anting are scattered around the bird. Photo: Octavio Jiménez Robles. Printed under a CC BY license, with permission from Octavio Jiménez Robles, original copyright 2012.
(PDF)
