# Supplementary material for: Non-foraging tool use in European Honey-buzzards: An experimental test
Source: PLoS One. 2018 Nov 21;13(11):e0206843. doi: 10.1371/journal.pone.0206843 (PMC6248935; doi:10.1371/journal.pone.0206843)
Supplement: S1 Fig — Focal bird of the study in central Spain, passing through the oak forest where maple and oak twigs were collected as a tool for anting. Photo: Octavio Jiménez Robles. Printed under a CC BY license, with permission from Octavio Jiménez Robles, original copyright 2012. (PDF) [file pone.0206843.s001.pdf]

Supporting information – Figure S1

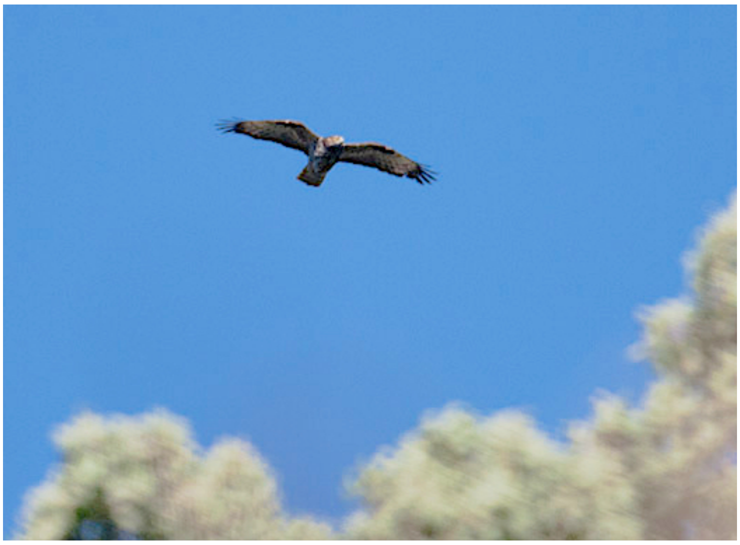

S1 Fig. European Honey-buzzard (*Pernis* apivorus). Focal bird of the study in central Spain, passing through the oak forest where maple and oak twigs were collected as a tool for anting. PHOTO: Octavio Jiménez Robles. Printed under a CC BY license, with permission from Octavio Jiménez Robles, original copyright 2012.
