# Supplementary material for: Non-foraging tool use in European Honey-buzzards: An experimental test
Source: PLoS One. 2018 Nov 21;13(11):e0206843. doi: 10.1371/journal.pone.0206843 (PMC6248935; doi:10.1371/journal.pone.0206843)
Supplement: S2 Fig — European Honey-buzzard (Pernis apivorus) standing on the ground, with partly spread wings and tail. Maple and oak twigs used as a tool to attract ants for anting are scattered around the bird. Photo: Octavio Jiménez Robles. Printed under a CC BY license, with permission from Octavio Jiménez Robles, original copyright 2012. (PDF) [file pone.0206843.s002.pdf]

Supporting information – Figure S2

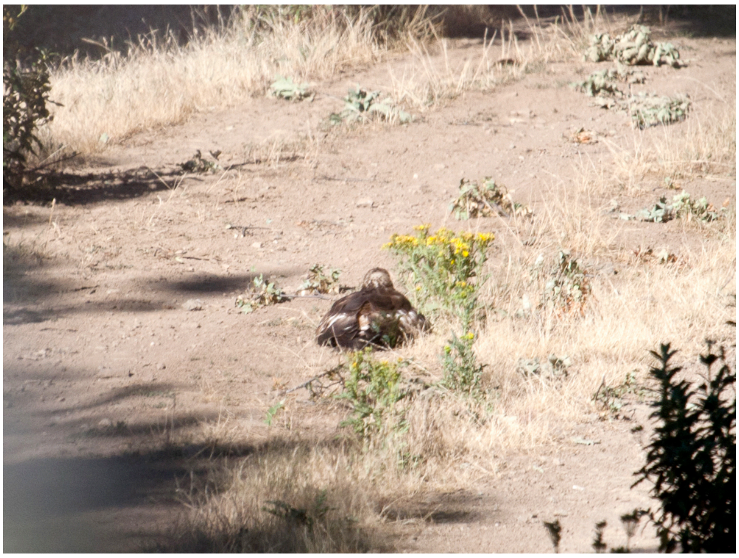

S2 Fig. Focal bird in a posture consistent with anting. European Honey-buzzard (*Pernis apivorus*) standing on the ground, with partly spread wings and tail. Maple and oak twigs used as a tool to attract ants for anting are scattered around the bird. PHOTO: Octavio Jiménez Robles. Printed under a CC BY license, with permission from Octavio Jiménez Robles, original copyright 2012.
